# Placebo response in binge eating disorder

**DOI:** 10.1002/eat.20287

**Published:** 2007-04

**Authors:** M Joy Jacobs-Pilipski, Denise E Wilfley, Scott J Crow, B Timothy Walsh, Lisa R R Lilenfeld, Delia Smith West, Robert I Berkowitz, James I Hudson, Christopher G Fairburn

**Affiliations:** 1 UCSD Eating Disorders Treatment CenterSan Diego, California; 2 Department of Psychiatry, Washington University School of MedicineSt. Louis, Missouri; 3 Department of Psychiatry, University of MinnesotaMinneapolis, Minnesota; 4 New York State Psychiatric Institute, Columbia-Presbyterian Medical CenterNew York, New York; 5 Department of Psychology, Georgia State UniversityAtlanta, Georgia; 6 Department of Health Behavior, College of Public Health, University of Arkansas for Medical SciencesFayetteville, Arkansas; 7 Department of Psychiatry, Weight and Eating Disorders Program, University of Pennsylvania School of Medicine, Philadelphia and Department of Child and Adolescent Psychiatry, The Children's Hospital of PhiladelphiaPhiladelphia, Pennsylvania; 8 Department of Psychiatry, Harvard Medical School and McLean HospitalBelmont, Massachusetts; 9 Department of Psychiatry, Oxford UniversityOxford, UK

**Keywords:** binge eating disorder, placebo response, eating disorders, psychopathology

## Abstract

**Objective:**

Placebo response in studies of binge eating disorder (BED) has raised concern about its diagnostic stability. The aims of this study were (1) to compare placebo responders (PRs) with nonresponders (NRs); (2) to investigate the course of BED following placebo response; and (3) to examine attributions regarding placebo response.

**Method:**

The baseline placebo run-in phase (BL) was part of a RCT investigating sibutramine hydrochloride for BED; it included 451 participants, ages 19–63, diagnosed with BED. Follow-up (FU) included 33 PRs.

**Results:**

In this study, 32.6% of participants responded to placebo (PRs = 147; NRs = 304). PRs exhibited significantly less symptom severity. At FU (*n* = 33), many PRs reported continued symptoms.

**Conclusion:**

PRs exhibited significantly less severe pathology than NRs. Placebo response in BED may transitory or incomplete. The results of this study suggest variable stability in the BED diagnosis. © 2006 by Wiley Periodicals, Inc. Int J Eat Disord 2007

## Introduction

Binge eating disorder (BED) consists of recurrent episodes of binge eating in which large amounts of food are eaten with accompanying loss of control.^1^Obesity is prevalent among individuals with BED.^2^–^4^ In recent years, there has been a noteworthy increase in pharmacotherapy studies for BED, primarily involving antidepressants, appetite suppressants, and anticonvulsant agents.^5^Results from these studies suggest a promising role for pharmacotherapy as a treatment component for BED.^5^

Placebo-controlled pharmacological studies of BED have revealed highly variable, and often marked, rates of short-term placebo response.^6^,^7^Placebo response in BED has been defined by a marked reduction in binge eating symptomatology during placebo administration. A review of pharmacological treatments for BED by Carter et al.^5^indicates a mean placebo response rate of 33%. The rate of placeboresponsein BED is similar to that in other major psychiatric illnesses, including major depressive disorder and bipolar disorder,^8^–^10^but not bulimia nervosa (BN), which has a markedly lower rate of placebo response than that associated with other psychiatric illnesses.^11^A meta-analysis of placebo response in unipolar depression indicated a mean placebo response rate of 32.8%.^9^A meta-analysis of bipolar disorder revealed a mean placebo response rate of 29%.^8^

Although placebo response occurs across a range of psychiatric diagnoses, investigation of the phenomenon in BED may provide increased clarity regarding the course and stability of the diagnosis and may be relevant to the presently unsettled nosological status of BED.^12^–^14^Rates of placebo response in BED suggest that there may be heterogeneity among ostensibly similar groups of patients. Fairburn and colleagues’^15^ natural course study of BN and BED found that after 5 years, only 18% of the BED participants still had an eating disorder, compared with 51% of the BN cohort. In contrast, preliminary results from the McKnight study reported by Crow et al.^16^ indicated a higher degree of stability of eating disorder symptoms in BED, with 93% of participants still having an eating disorder at 1-year follow-up. At issue is whether BED is a syndrome in need of active treatment or a condition that is likely to remit on its own.

To our knowledge, the present study is the first systematic investigation of placebo response in BED. The primary aims were to (1) compare pretreatment characteristics of placebo responders (PRs) and nonresponders (NRs); (2) to investigate the course of BED symptomatology among PRs following placebo response; and (3) to examine PRs’ attributions regarding the remission and/or return of their binge eating symptoms.

## Method

**FIGURE 1:**
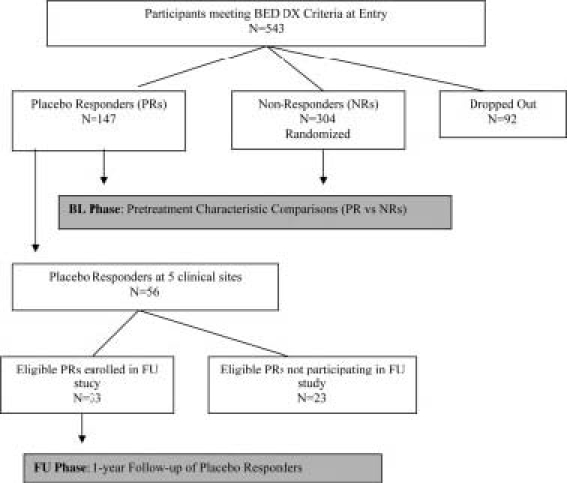
Study Design

This study occurred as part of a multicenter, randomized controlled trial to test the efficacy and safety of sibutramine hydrochloride for the treatment binge eating disorder (BED).^17^ Data collection took place in two phases, baseline (BL) and follow-up (FU), separated by approximately 1 year (1999–2000). BL data were collected as part of the sibutramine efficacy trial, which took place at 18 centers in the United States. From the 18 sites in the original study, a subset of five sites were elected to participate in a follow-up study. All research was reviewed and approved by an institutional review board. The primary aim of the FU study was to assess the course of eating disorder symptomatology among PRs approximately 1year post-exclusion from the treatment phase of the sibutramine trial (see Figure 1 for study design).

### BL Phase

#### Participants

All participants met full DSM-IV research criteria for BED.^18^ The diagnosis of BED was established using the Eating Disorder Examination (EDE),^19^ BED diagnostic version. Participants had to be between ages 18–65, with a body mass index (BMI) of <45 kg/m^2^. Exclusion criteria included an eating disorder (non-BED) within the previous 6 months, current use of psychoactive or weight loss agents or of medications potentially interfering with drug absorption, current insulin use, a history of alcohol or drug abuse (previous 12 months), a current psychiatric condition being treated with a psychoactive agent, major depressive disorder, and history of psychosis, bipolar disorder, or suicide attempts.^17^

Classification of placebo response occurred after 4 weeks of single-blind placebo administration. Individuals who continued to meet BED diagnostic criteria and who did not experience marked decreases in binge eating were included in the sibutramine trial. These participants were classified as placebo nonresponders (NRs). Participants who no longer met BED diagnostic criteria or who experienced a marked reduction in binge eating (total number of binge episodes for the previous week was 25% of that for the week preceding the beginning of the placebo run-in phase) were classified as PRs and were excluded from the sibutramine trial.

#### Measures

Eating disorder symptomatology was assessed at screening and FU using the BED diagnostic version of the EDE,^19^ an investigator-based, semi-structured interview designed to provide a complete assessment of BED. It is derived from the EDE 12.0D,^19^ which has well-established reliability and validity.^20^–^24^ It obtains a 6month history of objective binge eating episodes and other episodes of overeating, compensatory behaviors, and attitudes associated with binge eating (i.e., importance of weight and shape) and demonstrates good-to-excellent reliability in the assessment of BED.^25^

The EDE allows binge eating episodes to be categorized with respect to: (1) the amount of food eaten and (2) loss of control. Eating episodes that include an ‘‘unambiguously large’’ quantity of food (determined by the interviewer) and that occur with a loss of control are classified as objective bulimic episodes (OBEs). Eating episodes in which the interviewee reports a subjective sense of having overeaten (but are not objectively large) and that occur with a loss of control are classified as subjective bulimic episodes (SBEs). Occasions of eating unambiguously large amounts of food without a loss of control are classified as objective overeating (OO). Variables derived from the EDE included the average frequency of OBEs over the previous 6 months, as well as reported binge days and episodes (objective and subjective), OO days and episodes, and importance of weight and shape over the previous 28 days.

Bingeepisodeswererecordeddaily by participants for 4 weeks using a binge episode diary. Binge episodes were defined as ‘‘episodes of overeating during which you feel out of control’’.^17^ Participants recorded the time, content, ratings of loss of control (using a Likert-type scale), and distress related to all such episodes. These episodes were then evaluated in a clinical interview by an investigator using the overeating section of the EDE. Episodes determined to be OBEs were then recorded on case report forms.

Quality of life was measured using the Impact of Weight on Quality of Life Questionnaire (IWQOL-Lite).^26^ The IWQOL-Lite is a 31-item, self-report measure which demonstrates strong psychometric properties and assesses the impact of overweight status on quality of life, including five specific domains of quality of life: Work, Public Distress, Sexual Life, Physical Function, and Self-Esteem.^26^ Higher scores on the IWQOL-Lite indicate greater impairment.

#### Procedures

1. *Screening*: All participants provided written informed consent and received a physical exam, measurement of height and weight, and completed the EDE interview within the 2 weeks before placebo administration.^17^
2. *Placebo run-in (week-4 and week-2)*: Two visits occurred at 2-week intervals during the 4-week placebo administration period. Participants received a medical evaluation and submitted 2-week binge episode diaries for review. Any adverse medical events or medication changes were documented. At the first visit, participants completed the IWQOL-Lite and received a 4-week supply of placebo medication.
3. *Baseline visit (Day 1)*: All participants received a medical evaluation and returned 2-week binge episode diaries for review. Participants determined to be PRs were excluded from further participation in the sibutramine study; NRs not otherwise excluded were randomized.

#### Statistical Analysis I

All statistical analyses were conducted using Stata, version 9.1, or SPSS for Windows, version 11.5. Independent sample t tests were conducted to investigate pretreatment differences between PRs and NRs for each of the following variables separately: age, BMI, days and episodes of OBEs and SBEs for the preceding 28 days, average OBE days per week in the previous 6 months, OO days and episodes (previous 28 days), importance of weight (previous 28 days), importance of shape (previous 28 days), and quality of life (IWQOL-Lite total and subscale scores). The difference in gender was evaluated by Fisher’s exact test. Logistic regression was performed to investigate whether the EDE variables predicted PR status (controlling for age [categories for quintiles of the age distribution], gender, and BMI [categories: <30, 30–34, 35–39, and 40]); the values of the continuous predictor variables were scaled to SD units based on the sample distribution, so that the resulting odds ratio (OR) from logistic regression represented the OR for each 1 SD unit increase in the predictor. Alpha was set at .05, two-tailed.^a^

### Follow-Up (FU) Phase

#### Participants

PRs at participating sites were interviewed in person (or by phone if unavailable in person) approximately 1 year after study exclusion to assess eating disorder symptomatology, attributions regarding placebo response, and additional treatment obtained.

#### Measures

Eating disorder symptomatology was assessed using the BED diagnostic version of the EDE,^19^ modified to include assessment of all months following exclusion from the trial. PRs’ attributions for (1) initial placebo response and (2) subsequent remission and/or (3) return of eating disorder symptomatology were obtained through a three-question interview (available upon request) developed for this study. Participants could provide multiple attributions.

Based on a review of participant responses and the literature regarding the etiology and treatment of binge eating, the first author generated response categories for the classification of attributions by two trained, independent raters. Cohen’s kappa (k) was calculated to assess inter-rater agreement. With the exception of the ‘‘other’’ category, the classifications demonstrated moderate to excellent interrater reliability (k = .6–1.0). On items with imperfect agreement, the two raters (supervised by the first author) conferred to achieve consensus.

Data on additional treatment obtained were obtained by interview with study personnel.

#### Procedures

FU participants were interviewed approximately 1 year (range 8–18 months; m = 13.3 months, median = 12 months) following exclusion from the sibutramine trial. They completed the EDE and the attributional interview and also provided additional treatment information.

#### Statistical analyses II

To investigate sample representativeness, two-tailed independent sample t tests were used to compare the eating disorder symptomatology of the FU sample (at BL) with that of nonparticipating PRs. EDE variables included in these analyses were OBE days/episodes, SBE days/episodes, 6-month OBE average (days per week), OO days/episodes, importance of weight, and importance of shape.

Descriptive statistics of EDE variables were used to investigate the course of eating disorder symptomatology following placebo response. Frequencies were calculated for the following variables: OBE days (previous 28 days), SBE days (previous 28 days), and 6-month OBE average (days/week). The analytic approach taken to the attributional and additional treatment data was also descriptive. Alpha was set at .05, two tailed.^b^

## Results

### BL Phase

#### Participant Flow

**FIGURE 2:**
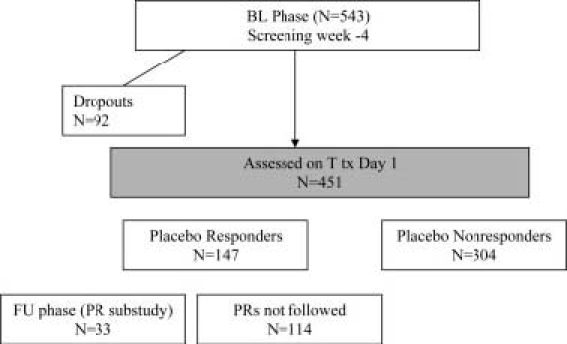
Participant Flow

Participants (n = 543) entered the single-blind placebo run-in phase at week-4; 92 dropped out of the study before baseline assessment (day 1). Of the remaining 451 participants, 147 (32.6%) were classified as PRs and 304 (67.4%) were classified as NRs (see Figure 2).

#### Participant Characteristics

PRs and NRs did not significantly differ with respect to age (p = 0.86), BMI (p = 0.10), or gender (p = 1.0). The mean (SD) for age was 41.8 (9.6) years, and BMI was 35.3 (5.3) kg/m^2^; 90.3% of subjects were female.

### Pretreatment Characteristics: PRs versus NRs

1. *Eating disorder symptomatology*: PRs and NRs significantly differed with respect to objective and subjective binge eating and overvaluation of weight and shape. Overall, PRs had significantly fewer OBEs on fewer days but reported more SBEs than did NRs. PRs reported significantly less emphasis on shape and weight in self evaluation than NRs (see Table 1). Cohen’s d effect sizes for the significant group differences were .3–.4.
2. Additional pretreatment differences:PRs reported significantly better overall and domain-specific quality of life (public distress, sex, and work subscales) than NRs (see Table 2). Cohen’s d effect sizes for the significant group differences were .2–.3.

**TABLE 1:** Pretreatment EDE comparisons

	PR (*n* = 147)	NR (*n* = 304)				
Variable	Mean (*SD*)	Mean (*SD*)	*t*	*df*^a^	*p* Value	Effect Size *d*
OBE days (previous 4 weeks)	14.9 (6.6)	16.9 (7.3)	2.83	446	0.005	0.3
OBE episodes (previous 4 weeks)	18.8 (11.2)	23.2 (13.7)	3.38	443	<0.001	0.4
6-month OBE average (days/week)	3.6 (1.7)	4.2 (1.6)	3.54	448	<0.001	0.4
SBE days (previous 4 weeks)	5.8 (8.4)	4.3 (7.5)	2.70	446	0.007	0.3
SBE episodes (previous 4 weeks)	8.1 (13.3)	4.3 (7.5)	1.88	447	0.061	0.2
Importance of weight (previous 4 weeks)	4.4 (1.4)	4.6 (1.3)	1.33	449	0.18	0.1
Importance of shape (previous 4 weeks)	4.5 (1.3)	4.9 (1.1)	2.99	449	0.003	0.3
OO^b^ days (previous 4 weeks)	1.9 (5.3)	1.9 (6.8)	0.05	447	0.96	0.0
OO episodes (previous 4 weeks)	1.9 (5.6)	2.0 (7.1)	0.16	447	0.87	0.0

Note: EDE = eating disorder examination; OBE = objective bulimic episode; SBE = subjective bulimic episode; OO = objective overeating; PR = placebo responder; NR = nonresponder; SD = standard deviation; df = degrees of freedom, d = standardized difference between 2 means.

a adf varies among variables due to missing data.

b Objective overeating: eating an unambiguously large amount of food with no accompanying loss of control.

**TABLE 2:** IWQOL-Lite ^a^ Pretreatment comparisons

	PR (*n* = 147)	NR (*n* = 304)				
IWQOL Scores	Mean (*SD*)	Mean (*SD*)	*t*	*df*^b^	*p* Value	Effect Size *d*
Total	70.9 (19.8)	76.4 (22.9)	2.38	420	0.018	0.3
Public distress	8.9 (3.8)	10.1 (4.4)	2.66	443	0.008	0.3
Sex	9.3 (4.1)	10.3 (4.6)	2.14	433	0.033	0.2
Work	7.7 (2.8)	8.5 (3.4)	2.45	439	0.015	0.3
Physical function	24.3 (8.6)	25.8 (9.9)	1.58	437	0.12	0.2
Self esteem	20.9 (6.8)	21.6 (7.1)	1.00	422	0.32	0.1

Note: PR = placebo responder; NR = nonresponder; *SD* = standard deviation; *df* = degrees of freedom.

a Higher scores on the Impact of Weight on Quality of Life Questionnaire (IWQOL-Lite) indicate greater impairment.

b *df* varies among variables due to missing data.

#### Prediction of PR Status

Logistic regression was conducted to investigate whether variables hypothesized to be related to the stability of BED predicted PR status. The five significant predictors of PR were the OBE days, OBE episodes, 6-month OBE average, SBE episodes, and importance of shape. For OBE days, OBE episodes, 6-month OBE average, and importance of weight, increasing values were associated with lower odds of PR, whereas with SBE episodes, an increasing value was associated with higher odds of PR. Specifically, for each 1 SD unit increase in the value of the predictor, the OR (95% confidence interval; P-value) for PR was: 0.75 (0.60–0.92; 0.006) for OBE days; 0.68 (0.54–0.86; 0.001) for OBE episodes; 0.69 (0.56–0.85; < 0.001) for 6-month OBE average; 1.30 (1.1–1.6; 0.012) for SBE episodes; and 0.76 (0.62–0.92; 0.007) for the importance of shape. SBE days, OO days/episodes, and importance of weight were not significant predictors.

### FU Phase

#### FU Participant Flow and Characteristics

Thirty-three PR subjects participated in the FU study, which represented 22.5% of the 147 total PR subjects and 58.9% of the 56 PR subjects eligible to participate in the five study centers conducting the PR study. Comparisons between PRs participating in the FU study (n = 33) and nonparticipating PRs (n = 133) indicate that the groups did not significantly differ with respect to any predictors of PR status (i.e., OBE days/episodes, 6-month OBE average, SBE episodes and importance of shape). The groups did differ, however, with respect to importance of weight. At study entry, the FU sample reported significantly lower importance of weight than the nonparticipating PRs. (mean [SD] of 3.8 [1.6] versus 4.1 [1.2], respectively; p = 0.001). Tests of the remaining variables revealed no other significant differences.

#### Eating Disorder-Related Symptomatology

At FU, 18 (54.5%) PRs were abstinent from OBEs in the previous month. Four PRs (12.1%) reported 1–3 OBE days in the past month, 8 (24.2%) reported 4–7 OBE days and 3 PRs (9.1%) reported 8 or more OBE days. Ten participants (30.3%) were abstinent from both OBEs and SBEs in the most recent month (see Table 3). Twenty-three participants (69.7%) reported SBEs in the past month. Over the previous 6 months, 56.3% of the sample reported OBEs on an average of 4 or more days per month; 35% reported diagnostic levels of binge eating (minimum average of 2 OBE days/week).

**TABLE 3:** Binge eating at follow-up

Binge Days (past 28 days)	Frequency (n)	%
0 OBEs	18	54.5
1–3 OBEs	4	12.1
4–7 OBEs	8	24.2
8þ OBEs 0 OBEs and 0 SBEs	3 10	9.1 30.3

Note: OBE = objective bulimic episode; SBE = subjective bulimie episode.

#### Additional Treatment.

Most of the FU sample (61%) received additional treatment during the follow-up period. Of treatment-seekers, 31.4 % (n = 10) obtained weight loss treatment, 27.3% (n = 9) received pharmacotherapy, 12.5% (n = 4) received individual psychotherapy, and 9.4% (n = 3) joined support groups. Thirty-five percent (n = 7) sought multiple treatments.

#### Relationship Between Abstinence from Binge Eating and Additional Treatment

Of the 18 participants abstinent from OBEs at month 1, 61.1% (n = 11) received additional treatment. Of the 10 participants who were OBE and SBE abstinent at month 1, 50% (n = 5) received additional treatment.

Obtaining additional treatment was the top reason cited by PRs for improvement in their binge eating symptoms.

#### PRs’ Attributions

1. *Initial placebo response*: FU PRs (*n* = 33) most frequently attributed their placebo response to increased awareness of their eating (43.8%), increased accountability to others (i.e., study personnel) (40.6%), self-monitoring (24.2%), social support (18.8%), and motivation to change (18.8%). Other responses included belief in the (placebo) medication, positive affect, positive expectations, being busy with other activities, and other/don’t know.
2. *Return of binge eating symptoms*: Most PRs (89%, n = 29) reported experiencing binge eating symptoms during the follow-up period. The most common attributions for the return of binge eating were stress (20.7%), negative affect (17.2%), and ‘‘other’’ (17.2%). Additional attributions included decreased motivation, personal failing, habit, decreased awareness, reduced accountability to others, reduced social support, increased food availability, discontinuation of self-monitoring, the nature of BED, lack of medication, and other/don’t know.
3. *Improvement in binge eating symptoms*: Participants who reported improvement in binge eating (n = 15; 45%) most commonly attributed it to receiving additional treatment (26.7%), being busy with other activities (20%), and health concerns (20%). Other responses included self-monitoring, social support, positive affect, motivation, appearance concerns, and other.

## Discussion

Similar to the rates reported for the controlled phases of other pharmacotherapy trials, approximately one third of the participants in the BL sample responded to placebo. With respect to eating disorder symptomatology and quality of life, PRs exhibited significantly less severe pathology than NRs. Nevertheless, within the time period between BL and FU, most of those PRs assessed at FU reported binge eating symptoms; more than one third met the BED diagnosis. PRs cited varied reasons for changes in their binge eating.

The 32.6% placebo response rate in this study is similar to mean placebo response rates reported in the treatment phases of trials involving other major psychiatric illnesses, including major depressive disorder^8^–^10^,^12^ and bipolar disorder.^8^,^27^,^28^ It is also consistent with the placebo response rate recently reported in the treatment phase of another trial investigating the use of sibutramine for BED^29^and to other placebo controlled trials for BED.

Comparisons between PRs and NRs reveal a consistent pattern of differences in eating disorder symptoms. NRs demonstrated significantly greater frequency and severity of OBEs than PRs and significantly more concerns about shape.

The importance of binge size with respect to the diagnosis of BED has been debated.^30^ PRs were significantly more likely to experience SBEs than NRs, but significantly less likely to have OBEs days or episodes. Binge size thus appears to discriminate participants who respond to placebo from those who do not. Logistic regression results suggest that a pattern of greater OBE days, OBE episodes, and 6-month OBE average, as well as a lower number of SBEs and higher importance of shape may reduce the odds of placebo response.

Obesity is highly prevalent among individuals with BED, affecting approximately 65% of individuals with BED.^3^Despite similar levels of obesity, the groups demonstrated significant differences with respect to its impact on quality of life. The data suggest that the deleterious impact of binge eating on quality of life in obese individuals is significantly greater than the negative impact of weight alone.^31^The poorer quality of life among NRs may be related to greater severity of their binge eating symptoms.

Although slightly more than one half of the FU sample reported no OBEs in the month immediately before the FU assessment, nearly 70% did report a loss of control over their eating. The significance of loss of control experiences among obese individuals with a history of BED requires further investigation.

With respect to the diagnostic status of the FU sample, approximately 35% of the participants experienced binge eating consistent with a BED diagnosis (2 OBE days/week for 6 months). Fifty-six percent of the participants experienced OBEs an average of at least 4 days per month over 6 months. Although this does not meet the diagnostic threshold for BED, data suggest that individuals with subthreshold BED (those whose binge size and/or frequency is below the diagnostic threshold) experience weight and shape concerns, psychiatric distress, and seek treatment for their eating and weight problems at rates similar to those of individuals diagnosed with BED.^32^

Potentially indicative of participants’ continued distress over their eating is the considerable percentage (61%) that obtained additional treatment. The treatments most frequently obtained were behaviorally-based weight loss treatment and pharmacotherapy (e.g., sibutramine).

Participants retrospectively attributed their placebo response to personal factors (such as increased awareness of their eating behavior, self-monitoring, increased motivation to change their eating behavior) and interpersonal factors (having to be accountable to others, social support). The most commonly cited reasons for the resumption of binge eating were environmental (e.g., stress) and personal (such as depressed mood, decreased motivation, sense of personal failing, decreased awareness of eating behavior, habit). Reasons cited for continued improvement following the initial placebo response were largely environmental (e.g., obtaining additional treatment, participating in other activities) and personal (i.e., health concerns, self monitoring, positive affect, high motivation, appearance concerns).

Several limitations of the present study should be considered. Nonspecific treatment effects (i.e., contact with study personnel) potentially confound the measurement of placebo response,^10^ as do other factors, such as natural course of illness, expectation effects, and regression to the mean.^8^–^10^,^28^,^33^ Similar to other pharmacological studies of BED, the study protocol asked all participants to monitor their eating behavior during the study. The self-monitoring component of the present study was designed to reduce reactivity (e.g., both groups self-monitored); nevertheless, the act of self-monitoring may have affected participants’ eating behavior. These data may also have been inﬂuenced by selection bias; all participants sought treatment and consented to participate in a placebo-controlled trial, potentially indicating expectation effects regarding placebo. Because the assessment of initial PR was coded categorically (PR or not PR), no data on percentage reduction in binge eating following initial PR are available. FU participants consented to be reinter-viewed and may have experienced superior outcomes compared with PRs who were not available for follow-up assessment. The entry criteria for the study (BMI <45 kg/m^2^, no comorbid disorders) may have excluded participants with more severe psychopathology and poorer prognoses. Finally, additional participants may have responded to placebo during the subsequent double-blind phase; examination of these individuals could potentially elucidate the fuller range of placebo response in BED. Although the FU sample appears representative with respect to predictors of placebo response, a larger sample assessed at discrete intervals is required to increase the inferential power of these findings.

## Conclusion

Use of a placebo control has become the sine qua non of the randomized controlled trial.^33^ Individuals with BED who respond to placebo appear to demonstrate less severe eating disorder psychopathology and less impairment in quality of life than do nonresponders (NRs) and may potentially benefit from briefer, less intensive interventions than NRs. Short-term intervention with a placebo, however, appears of little value with respect to the long-term management of these binge eating problems. Even among individuals with fewer complications related to obesity and comorbid psychopathology, BED may be a refractory condition.

The authors are grateful to Therese Gregory-Bills for her participation in this project

## Footnotes

a Given the exploratory nature of the analyses, corrections for multiple comparisons were not performed.

b Given the exploratory nature of the analyses, corrections for multiple comparisons were not performed.
